# Oral and Dental Abnormalities Caused by a Pediatric Rhabdomyosarcoma Tumor Treatment: A Clinical Case Report

**DOI:** 10.3390/dj8020059

**Published:** 2020-06-18

**Authors:** Luísa Bandeira Lopes, Rodrigo Themudo, João Botelho, Vanessa Machado

**Affiliations:** 1 Pediatric Department, Centro de Investigação Interdisciplinar Egas Moniz, Egas Moniz Cooperativa de Ensino Superior, 2829-511 Caparica, Almada, Portugal; 2 Clinical Research Unit (CRU), Centro de Investigação Interdisciplinar Egas Moniz (CiiEM), Instituto Universitário Egas Moniz (IUEM), 2829-511 Caparica, Almada, Portugal; rodtgm_11@hotmail.com; 3 Periodontology Department, Clinical Research Unit (CRU), Centro de Investigação Interdisciplinar Egas Moniz (CiiEM), Instituto Universitário Egas Moniz (IUEM), 2829-511 Caparica, Almada, Portugal; joaobotelho09@gmail.com (J.B.); vanessamachado558@gmail.com (V.M.)

**Keywords:** rhabdomyosarcoma, oral pathology, pediatric dentistry

## Abstract

Rhabdomyosarcoma is one of the most common soft-tissue sarcomas in children. The therapy for this condition has evolved significantly over recent decades, as has survival rates. Nevertheless, multiagent chemotherapy, radiation therapy, surgical resection or a combination of these modalities still have to be performed. This case report presents a 16-year-old boy with oral and dental effects after rhabdomyosarcoma treatment, diagnosed at the age of 4 years old. This report highlights the key role of dentists in the clinical management of rhabdomyosarcoma cases before, during and after treatment, and its potential side effects.

## 1. Introduction

Rhabdomyosarcoma (RMS) is a malignant neoplasm with skeletal muscle differentiation. It is the most common soft tissue sarcoma in childhood and is most frequently located in the head and neck [1,2,3,4,5,6,7,8]. Weber first described RMS in 1854, as highly aggressive malignant neoplasm [9]. Even so, the diagnosis of RMS has always been a challenge, due to its varied clinical presentation and histological diversity, and its exact etiopathogenesis remains unknown [9]. During the past years, the World Health Organization (WHO) classification of RMS has suffered several changes. The first Intergroup Rhabdomyosarcoma Study started in 1972, where the high mortality rate was highlighted [10].

Currently, WHO classifies RMS into embryonal, alveolar, spindle/sclerosing, and pleomorphic subtypes and does not distinguish the botryoid subtype, since the RMS embryonal is the most common [2,4,5,7,11,12,13,14]. Although the etiology and environmental factors of RMS are still unclear, some genetic alterations have been recently outlined in the pathogenesis of RMS [1].

A correct and early diagnosis of RMS in childhood is key for the clinical outcome. Overall, an appropriate diagnosis includes histologic subtype, primary site and stage, so that a prompt treatment can be performed [3,11,15,16]. The treatment approaches depend on several parameters (such as the site, the staging and extent), therefore the tailored therapeutic approach depends on these criteria [16]. 

The prognosis and treatment course in RMS also depend on the anatomical location (orbital, parameningeal or non-parameneningeal). Regarding the type of RMS, the embryonic type is more common in younger patients, but in turn the incidence decreases with age [17].

Although RMS treatment approaches have progressed in recent decades, the multimodal treatment is still the most usual choice [2,6]. Comprehensively, chemotherapy based on a cocktail of ifosfamide, vincristine and actinomycin, radiation therapy and surgical resection continues to be common practice [18,19,20,21,22,23,24,25]. A recent consensus approach to RMS highlighted recent findings, as well as identified areas in need of further research [26]. The complexity of the RMS makes it necessary to underline the need for a structured approach, with patients benefiting from a multidisciplinary and adjusted service [26].

Reports of late oral effects after multimodal treatment, at an early age, have been mentioned since 1970s, indicating the interruption of jaw growth [27]. Later, there were other reports referring to impacted teeth, microdontia and teeth with underdeveloped roots [10].

Thus, combined modality treatment has been described to cause late oral effects, particularly when performed at a young age. When a multi modal treatment is accomplished, it is important to understand how it can interfere with dental development, salivary gland conditions, and endocrine disorders among other disabilities [15,23,28].

The aim of this clinical report is to describe a 16-year-old boy with late oral defects after multimodal treatment of RMS at the age of 4 years old. This case aims to highlight how important the role of dental practitioners in RMS cases is, particularly because of the secondary oral and dental side effects of multimodal treatment.

## 2. Case Presentation

A 16-year-old male patient presented at a private clinic in Portugal for a dental routine check-up. Informed consent was obtained from his parents so that case records could be made available for teaching purposes, including scientific publication. All procedures followed the Helsinki Declaration, as reviewed in 2013.

### 2.1. Medical History

The patient was diagnosed at the Institute of Oncology of Lisbon (Portugal) with Embryonal Parameningeal Rhabdomyosarcoma at the age of 4 years old, which was located at the base of the cranial temporal fossa and without metastases. 

The tailored treatment combined a chemotherapeutical cocktail of Ifosfamide and Doxorubicin, as well as radiotherapy at 45 Gy on the tumor site. Finally, it was recommended left nasosinusal endoscopic surgery with left infundibulectomy, sphenoidectomy and exeresis of the pterygopalatine region. The pathology results confirmed the final diagnosis of RMS.

During the first cycle of chemotherapy, the patient presented generalized tonic-clonic convulsions as well as amaurosis of the left eye, with an irreversible tumor lesion of the optic nerve, since the tumor reached the great wing of the sphenoid and the vertex of the left orbit, and finally rupture of the tympanic membrane on the right side.

### 2.2. Physical Examination

**Figure 1:**
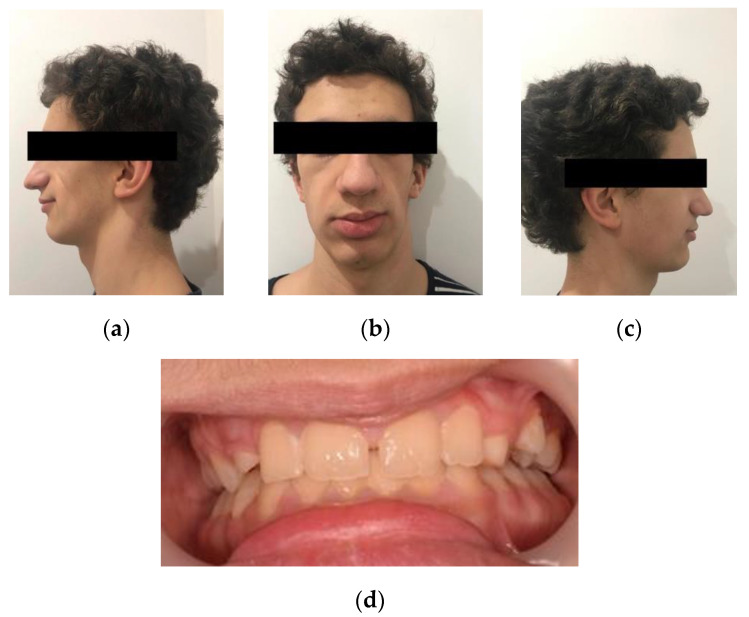
Extra-oral and intra-oral frontal photography. (**a**) Left side; (**b**) Frontal; (**c**) Right side; (**d**) Frontal intra-oral photography.

Patient presents growth hormone deficiency, amaurosis on the left eye, right tympanic rupture, but with good auditory acuity and facial asymmetry due to left hemiface hypoplasia (Figure 1). For these, current medication comprises Somatropin (1.7 mg at night), Levothyroxine (50 mg in the morning), and Hydrocortisone (10 mg, being half a tablet in the morning and the other half at night). 

According to the patient, his main complaints are the dysgeusia, with prevalence of a metallic taste, and total loss of vision of the left eye. 

### 2.3. Intra-Oral Examination

**Figure 2:**
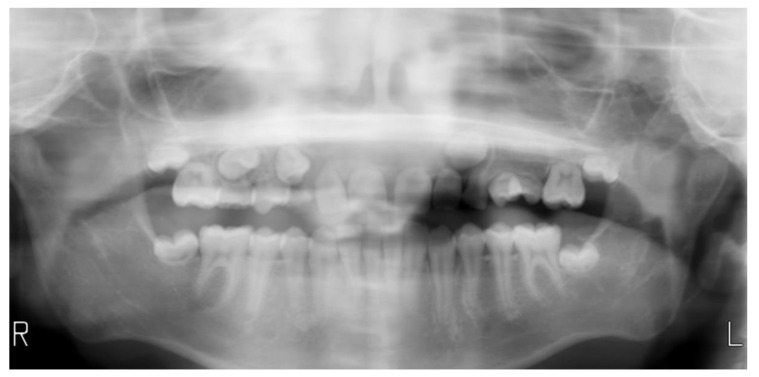
Panoramic X-ray.

**Figure 3:**
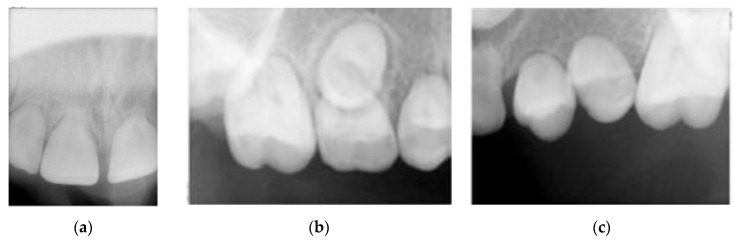
Periapical upper arch X-rays. (**a**) Teeth 1.1 (left) and 2.1 (right); (**b**) First quadrant with 1.4, 5.5 and 1.6 roots missing; (**c**) Second quadrant with 2.4, 2.5 and 2.6 roots missing.

**Figure 4:**
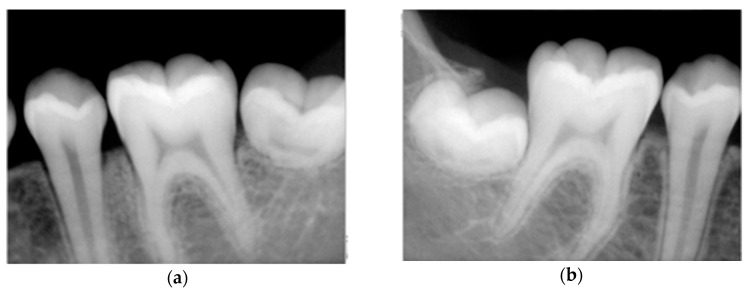
Periapical lower arch X-rays. (**a**) Third quadrant with 3.5, 3.6 and missing roots of 3.7; (**b**) Fourth quadrant with 4.5, 4.6 and missing roots of 4.7.

Intra-oral examination showed a good oral hygiene and no dental cavities (Figure 1). The teeth in the lower arch, and incisors and first molar in upper arch appeared to be of normal shape, size and appearance. However, panoramic X-ray (Figure 2) revealed several teeth with abnormalities at the root level. This incomplete root development, also known as root stunting, affected all the teeth in the maxilla (Figure 3) and teeth 37 (Figure 4a) and 47 (Figure 4b) in the mandible. Despite these radiographic findings, the affected teeth showed no mobility. It also observed some retained primary teeth on the upper arch with the corresponding permanent not erupted (Figure 3b). 

Due to these findings and since the teeth were functional and there was no sign of pulp involvement, no further dental treatments were necessary. However, the patient was informed that it is crucial to maintain good oral hygiene and to have periodic appointments in order to avoid any further complication and even the precocious loss of the teeth.

Considering the presented clinical status, the patient was advised to seek regular oncology and endocrinology appointments. Additionally, annual ophthalmology follow-up and regular cardiology follow-up for monitoring of cardiac function are mandatory, with ultrasound and electrocardiogram readings being recommended every 5 years.

## 3. Discussion

This case reports an abnormal root development due to a history of embryonal parameningeal RMS and treatment at the age of 4 years old. The underdevelopment of the roots of the teeth may have occurred due to the RMS treatments. In this case, root formation has been abruptly stopped at similar developmental stages for the maxillary and mandibular teeth, apparently because of highly aggressive treatment such as the RMS therapeutic.

The treatment of RMS consists of a combination of chemotherapy with additional surgery and/or radiation therapy. This potential risk of this multimodal treatment with high doses of toxicity are well established, and late effects are a highly prevalent [18]. As such, one of the potential risks is odontogenesis impairment, where the activity of odontoblasts is inhibited, and indirectly affects amelogenesis by inducing formation of “steodentin” which replaces normal dentin [29]. Moreover, according to the dose spectrum of the radiation therapy, tooth development is interrupted by 30 Gray (Gy). However, dental late effects have also been at radiation doses of only 4 Gy [29,30]. Furthermore, the radiation therapy doses depend on numerous factors, such as patient age, total radiation dose, exposed tissue volume, daily radiation, interaction with chemotherapy agents, and the development stage at the time of cancer treatment [25,29,30].

Under these considerations, the observed roots underdevelopment was due to the radiation therapy that this young patient has undergone. Despite the consensus about the presence of root stunting, it is not possible to identify an exact prevalence [18,29,30,31]. Kaste et al. [32] described 22 children with RMS submitted to radiotherapy and chemotherapy and found root stunting in 12 patients, while Sonis et al. [33] found a root stunting prevalence of 100% in 20 patients, and Maciel et al. [34] reported that 12 cases in 56 survivors had root stunting. Regarding the retention of primary dentition, this condition, despite being referred by some authors, has a lower prevalence than root stunting [29,35].

In a cross-sectional study of 88 children submitted to treatment to RMS, between the ages of 0 and 5 years, De Mattos et al. refer to several dental abnormalities such root stunting, and partial and total anodontia. Accordingly, the upper second molars were the most affected, followed by upper left canines [36].

Nevertheless, the reader must be aware that this radiation treatment can lead further to other dental abnormalities such as dental agenesis, dental hypoplasia, root stunting, hypodontia, enamel hypoplasia and microdontia [24,28,29,30,31]. Further, high doses of radiotherapy can also develop craniofacial abnormalities that can have impact in facial asymmetry [24,28,30]. 

On the other hand, radiotherapy may inevitably target other regions, such as the hypothalamic pituitary, due to their close anatomical relationship. Therefore, endocrine disorders are a possibility, such as growth hormone deficiency as reported in this case. Moreover, thyroid-stimulating hormone deficiency, adrenocorticotropic hormone deficiency, and gonadotropin deficiency can be diagnosed in RMS cases [21]. Importantly, salivary glands can be damaged, which turn saliva more acidic, and can contribute to a more cariogenic oral microflora [23,29], altering taste sensibility (like ageusia, hypogeusia and dysgeusia due to direct radiation on the taste buds). Usually, after radiation therapy ends, the acuteness of taste is restored but some patients may experience irreversible changes or even loss of taste [16,23].

Additionally, RMS survivors are also at more risk to develop caries lesions due to lower salivary flow rate and dental abnormalities secondary to chemotherapy. However, this patient presented great hygiene behaviors and no caries events.

It is also important to emphasize the likelihood of osteoradionecrosis (ORN) events in patients previously treated with radiotherapy [37]. ORN of the jaw is a severe complication of head and neck radiation therapy and the prevalence varies between 2% and 18% [38]. Bone trauma and particularly post-radiation extractions are a critical risk factor for ORN development, and the risk increases with higher radiation doses, greater than 60 Gy [39,40,41]. Patients requiring teeth extractions more than 5 years after radiotherapy have 16.5% of risk of ORN [41]. In this case, the severe roots stunting may jeopardize the clinical stability of teeth if any oral disease occurs, such as caries or periodontitis. If any of these illnesses initiate and are unresolved, this patient will need teeth extraction and may be at risk of developing ORN. Therefore, preventive measures are key to balance the oral health of this patient throughout his life.

Given the complications and side effects that multimodal treatment of RMS can lead to, the role of the dentist is crucial before, during and after oncologic treatment [16]. 

In fact, Dentists, Orthodontists, Dentomaxillofacial Radiologists and Dentomaxillofacial Surgeons involved in oral care must be able to recognize, diagnose and be prepared for the various late dental effects [25].

Overall, before starting cancer therapy it is advised to do X-rays to detect possible current problems such as dental caries. During treatment, recurrent fluoride applications are important to cause remineralization of the enamel and, thus, to prevent caries. After eradication of the malignant tumor, panoramic radiographs are recommended for reassessment and to consider root development if orthodontic treatment is necessary [16,29,30]. 

In conclusion, this article underlines a severe case of secondary oral complications of RMS treatment. Thus, we emphasize the importance of dental practitioners and the even higher need for appropriate oral health care in this particular case. Afterwards, an appropriate oral management will guide the parents and the child to a better quality of life.
